# Supplementary material for: Higher vascularity at infiltrated peripheral edema differentiates proneural glioblastoma subtype
Source: PLoS One. 2020 Oct 14;15(10):e0232500. doi: 10.1371/journal.pone.0232500 (PMC7556526; doi:10.1371/journal.pone.0232500)
Supplement: S1 Appendix — (DOCX) [file pone.0232500.s004.docx]

**S1 Appendix. Mann-Whitney U-test comparing rCBV_max_ at each habitat for Verhaak subtypes**

**Table S1.1.** Mann Whitney U-test p-values comparing (1) rCBV values at HAT habitat in each subtype against the others individually and (2) rCBV values of each subtype against the rest.

| rCBVmax HAT | Classical | Mesenchymal | Neural | Proneural |
| --- | --- | --- | --- | --- |
| Classical | 1 | - | - | - |
| Mesenchymal | 1 | 1 | - | - |
| Neural | 1 | 0.8573 | 1 | - |
| Proneural | 0.8573 | 0.4878 | 0.8573 | 1 |
| Rest | 1 | 0.8185 | 1 | 0.7117 |

All p-values are multiple test corrected

**Table S1.2.** Mann Whitney U-test p-values comparing (1) rCBV values at LAT habitat in each subtype against the others individually and (2) rCBV values of each subtype against the rest.

| rCBVmax LAT | Classical | Mesenchymal | Neural | Proneural |
| --- | --- | --- | --- | --- |
| Classical | 1 | - | - | - |
| Mesenchymal | 0.9743 | 1 | - | - |
| Neural | 0.7971 | 1 | 1 | - |
| Proneural | 0.2053 | 0.2053 | 0.2269 | 1 |
| Rest | 0.5180 | 0.7971 | 1 | 0.2053 |

All p-values are multiple test corrected

**Table S1.3.** Mann Whitney U-test p-values comparing (1) rCBV values at LAT habitat in each subtype against the others individually and (2) rCBV values of each subtype against the rest.

| rCBVmax VPE | Classical | Mesenchymal | Neural | Proneural |
| --- | --- | --- | --- | --- |
| Classical | 1 | - | - | - |
| Mesenchymal | 0.7773 | 1 | - | - |
| Neural | 1 | 0.3618 | 1 | - |
| Proneural | 0.3970 | 0.0492^*^ | 0.2017 | 1 |
| Rest | 1 | 0.1213 | 1 | 0.0619 |

All p-values are multiple test corrected; * for statistical significance
