# Supplementary material for: Higher vascularity at infiltrated peripheral edema differentiates proneural glioblastoma subtype
Source: PLoS One. 2020 Oct 14;15(10):e0232500. doi: 10.1371/journal.pone.0232500 (PMC7556526; doi:10.1371/journal.pone.0232500)
Supplement: S2 Appendix — (DOCX) [file pone.0232500.s005.docx]

**S2 Appendix. Cox proportional hazards regression for rCBV_max_ at ET and Verhaak subtypes**

**Table S2.1.** Cox proportional hazards regression results for a total of 9 models: univariate using rCBV_max_ at IPE and Verhaak subtypes and multivariate using their combination.

|  | **rCBV_max_ ET** | | **Verhaak Subtype** | |  |
| --- | --- | --- | --- | --- | --- |
|  | **HR (CI95)** | **p-value** | **HR (CI95)** | **p-value** | **AUC** |
| **rCBV_max_ ET** | 1.11 (1.0, 1.2) | 0.0102^*^ | - | - | 0.5611 |
| **Classical** | - | - | 0.99 (0.5, 2.0) | 0.9748 | 0.5037 |
| **Mesenchymal** | - | - | 0.83 (0.4, 1.6) | 0.5904 | 0.5407 |
| **Neural** | - | - | 0.61 (0.3, 1.3) | 0.1958 | 0.5403 |
| **Proneural** | - | - | 2.58 (1.2, 5.4) | 0.0113^*^ | 0.5847 |
| **rCBV_max_+ Classical** | 1.11 (1.0, 1.2) | 0.0091^*^ | 0.89 (0.4, 1.9) | 0.7614 | 0.5713 |
| **rCBV_max_+ Mesenchymal** | 1.11 (1.0, 1.2) | 0.0111^*^ | 0.85 (0.4, 1.7) | 0.6491 | 0.5602 |
| **rCBV_max_+ Neural** | 1.10 (1.0, 1.2) | 0.0155^*^ | 0.66 (0.3, 1.4) | 0.2882 | 0.5787 |
| **rCBV_max_+ Proneural** | 1.11 (1.0, 1.2) | 0.0167^*^ | 2.42 (1.2, 5.1) | 0.0185^*^ | 0.5981 |

^*^ for statistical significance.
