# Supplementary figures and images for: Higher vascularity at infiltrated peripheral edema differentiates proneural glioblastoma subtype
Source: PLoS One. 2020 Oct 14;15(10):e0232500. doi: 10.1371/journal.pone.0232500 (PMC7556526; doi:10.1371/journal.pone.0232500)

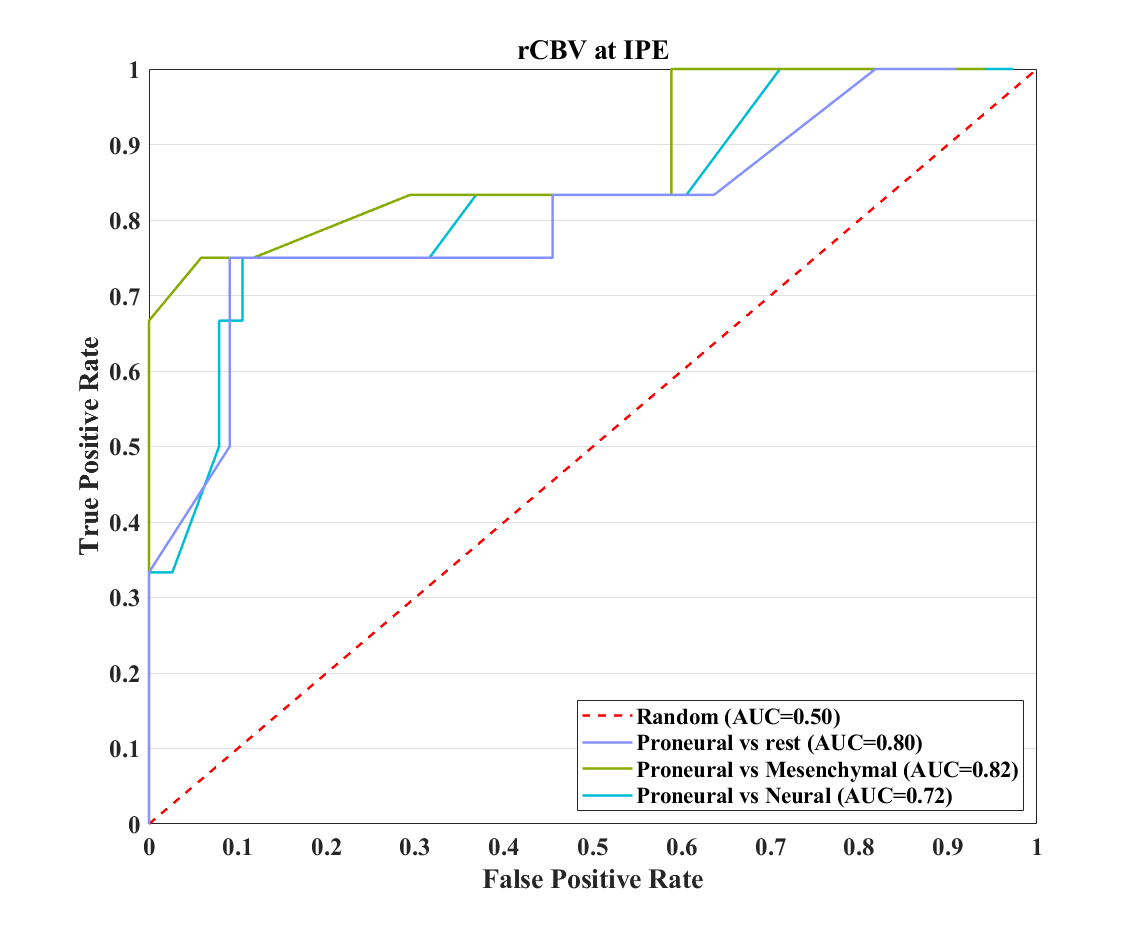

Supplement: S1 Fig — Significant experiments: distinguishing proneural from mesenchymal, proneural from neural and proneural from the rest. (TIF) [file pone.0232500.s006.tif]
